# Radionuclides (^210^Po and ^210^Pb) and Some Heavy Metals in Fish and Sediments in Lake Bafa, Turkey, and the Contribution of ^210^Po to the Radiation Dose

**DOI:** 10.3390/ijerph13111113

**Published:** 2016-11-09

**Authors:** Ramazan Manav, Aysun Uğur Görgün, Işık Filizok

**Affiliations:** Institute of Nuclear Sciences, Ege University, Izmir 35100, Turkey; aysun.ugur@ege.edu.tr (A.U.G.); ifilizok@gmail.com (I.F.)

**Keywords:** ^210^Po, ^210^Pb, fish, sediment, Lake Bafa, heavy metals

## Abstract

The pollution level of Lake Bafa was investigated by collecting fish samples {*Dicentrarchus labrax* (sea bass), *Liza ramada* (mullet) and *Anguilla anguilla* (eel)}, surface sediment, and core samples. In all these samples, ^210^Po and ^210^Pb concentrations were estimated, and total annual dose rates were obtained for each species. Some heavy metal (Cr, Ni, Pb, Cd, Mn, Fe, and Zn) concentration levels were obtained for the fish and a core sample. The sediment mass accumulation rate was found to be 3.27 g·m^−2^·day^−1^ (0.119 g·cm^−2^·y^−1^) from a core sample. The heavy metal concentrations in the vertical profile of samples from the core were also observed. The measured concentration of Zn, Pb, Cd, and Cr were between the ERL (effects range low) and ERM (effects range median) limits, while Ni concentrations were higher than the ERM limit. The observed concentrations of Cd, Pb, and Zn in fish samples did not exceed the limits in accordance with Turkish Food Regulations. Further, the maximum effective dose equivalent of ^210^Po in the area was found to be 1.169 µSv·y^−1^.

## 1. Introduction

Wetlands are an important source of global biodiversity and have been threatened by natural and human activities [1]. Extensive literature shows that lakes are suffering from contamination of metals and radionuclides. Lake ecosystems have been contaminated from anthropogenic sources. They may contain heavy metals from various sources such as urban garbage, urban fugitive, and automobile exhaust [2]. Several metals such as Zn, Co, and Cu are necessary elements for life; on the other hand, Pb and Cd are toxic to a great variety of habitats. Moreover, the excess usage of all metals can have a toxic effect to an organism [3]. ^210^Po and ^210^Pb are well-known natural radionuclides and are absorbed by plankton in aquatic environments. These radionuclides are accumulated by marine organisms and transferred to people with ingested food [4]. The activities of these two elements may be increased by industrial waste and human activities. 

The main aim of this study was to determine the concentrations of certain metals and radionuclidies in Lake Bafa by collecting biota and sediments. Biota samples {*Dicentrarchus labrax* (sea bass), *Liza ramada* (mullet), and *Anguilla anguilla* (eel)} and surface sediments were gathered monthly and seasonally, respectively. In addition, a core sample was obtained from the lake to get information about the activities of radionuclides and metal contents in recent years. 

The pollution of the lake is very important for people and the habitat of the lake. Natural pollution is assisted by human activities. The Menderes River, the water source of Lake Bafa, has many industrial factories down the river. In addition, the lake is covered by vulnerable agricultural soil. All of these increase the level of pollution of the lake because of unsustainable source use, illegal fishing, factory wastes and excessive use of water of the river for agriculture. Singare et al. [5] stated that among the different water bodies, lakes have a complex and fragile ecosystem, as they do not have self-cleaning ability and therefore readily accumulate pollutants.

## 2. Materials and Methods

### 2.1. Study Area

**Figure 1:**
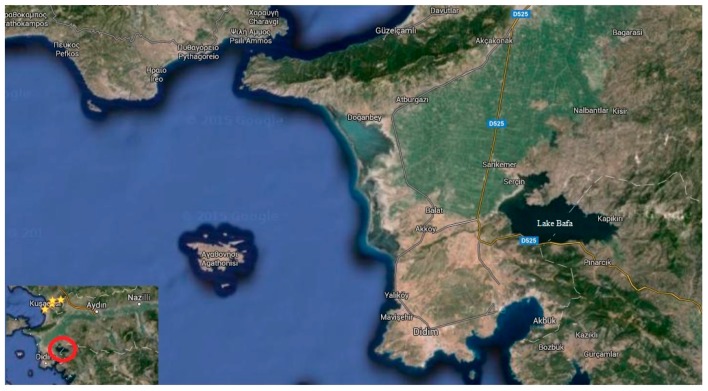
General view of Lake Bafa [7].

Lake Bafa is located in the southwestern part of Turkey, at the boundaries of Muğla and Aydın, at the coordinates 37°30′ N and 27°25′ E, as shown in Figure 1. The lake is mostly shallow, with a maximum depth of 21 m and a surface area of approximately 7500 ha. It is a delta lake formed by the Büyük Menderes River. The sediments of the river filled the marine, the Latimen Gulf, over the past six or so millennia; thus, it is separated from the sea [6]. Because of the ancient location area and the habitat of the lake, it has been legally protected as a natural park since 1994. In the villages around the lake, agriculture and fishing still occupy an important part of their economies.

### 2.2. Sampling

To estimate the pollution level of the lake, certain fish samples, surface sediments, and core sample were collected. The most consumed fish samples were *Dicentrarchus labrax* (sea bass), *Liza ramada* (mullet), and *Anguilla anguilla* (eel), and surface sediments were collected monthly and seasonally, respectively. Surface sediments were collected by Ekmann Grab, and the core sample was obtained by a gravity core-sampler. Ekmann Grab collected the upper sediment layer, with a thickness varying from 4 to 20 cm, depending on the bottom hardness. By using all these samples, ^210^Po and ^210^Pb concentrations were estimated, and total annual dose rates were obtained for each species. Certain heavy metal (Cr, Ni, Pb, Cd, Mn, Fe, and Zn) concentration levels were obtained for fish and core samples.

**Figure 2:**
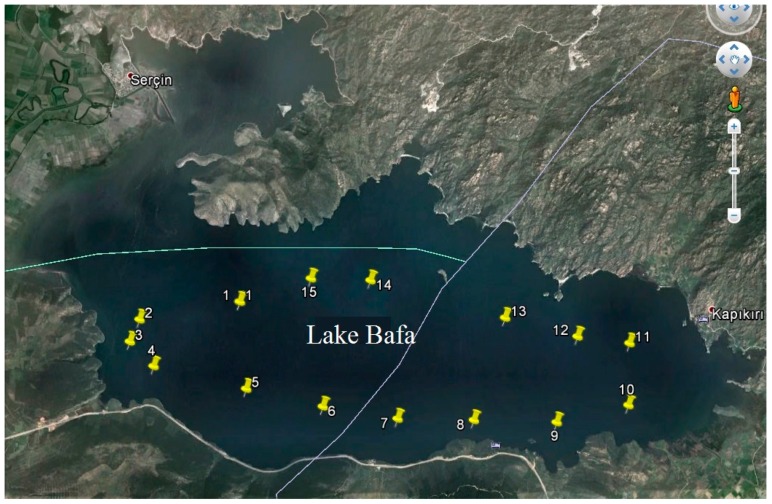
Sampling points of surface sediments [8].

The fish species were bought from fishermen of Lake Bafa in the Serçin Village in 2012. In the summer months, collecting fish in the lake is prohibited; therefore, there are no samples for the summer. The surface sediments were collected from about 8 cm deep in the middle of each season. The points where the surface sediment samples were collected are shown in Figure 2. The core samples were collected from the middle of the lake.

The muscles of the biotas were separated with a plastic knife. The muscle tissues were dried in an oven at 80 °C until they reach a constant weight. A core sample was cut into 6 mm long pieces for the first 10 cm and then into 12 mm long pieces until 33 cm. Core and surface samples were dried at 70 °C until a constant weight level was maintained. All samples were passed through a 250 μm mesh sieve for homogenization.

## 3. Laboratory Analysis

### 3.1. Alpha Spectrometric Analysis for ^210^Pb

Measurements of ^210^Po were realized through its 5.30 MeV alpha particle emission, using ^209^Po (4.88 MeV, *t*_1/2_ = 109 y) as the internal tracer. After a standard addition of polonium tracer, each sample was dissolved using three portions of concentrated 20 mL HNO_3_ and then 2 mL H_2_O_2_ evaporated to near dryness on a hot plate. To the remainder, three portions of 20 mL HCl were added, and the solution was evaporated to dryness. For sediment samples, HF was also used in the dissolving process. Finally, polonium was plated on a copper disc from a dilute HCl medium in the presence of ascorbic acid to reduce Fe^3+^ to Fe^2+^. Ortec Octete Plus with a 450 mm^2^ ULTRA-AS detector was used to measure the alpha activities. Concentrations of ^210^Po in all samples were well above the detection limit (0.0003 Bq). Sample count times were 86,400 s, with counting errors in the order of ±10% or less.

After the first deposition of ^210^Po, the residual 0.5 M HCl was kept for one year to allow ^210^Po in-growth from the ^210^Pb contained in the solution to search supported ^210^Pb in the samples. The samples were re-plated, and the ^210^Po activities were determined. Well-known Bateman equations were used to determine ^210^Pb activity from measured ^210^Po activity [9]. The second deposition provided information on the ^210^Pb content of the samples and hence on the extent to which the initial ^210^Po was supported by its grandparent.

### 3.2. Dose Calculations

Due to the dense ionization in tissues caused by high-energy alpha particles, ^210^Po has long been recognized as an important contributor to the radiation dose received by organisms [10]. For the calculation of dose due to ^210^Po, the following formula was used [11].

(1) $$D_{Po}\left(fish\right)=C_{b}F_{c}F_{h}D_{f}4.3\times 10^{-7},$$

where *D_Po_* is the collective committed effective dose of ^210^Po via consumption of fish from intake during the sampling period. The unit of collective committed effective dose is (man Sv). The coefficients *C_b_* and *F_c_* denote activity concentration of ^210^Po in the edible part of the fish samples (Bq·kg^−1^) and the catch calculated from Turkish Statistical Institute (TURKSTAT) statistics (kg·y^−1^), respectively. *F_h_* is the fraction of the catch that is used for regional human consumption (assumed to be 0.7), *F_e_* is the fraction that is actually eaten (assumed to be 0.8), *D_f_* is the delay factor between the catch and consumption time, and 4.3 × 10^−7^ is the factor used for adult members of the public, the recommended dose conversion coefficient [12].

### 3.3. Heavy Metal Analysis

The microwave method was applied for the digestion procedure of samples. Dried biota samples of 0.5 g were placed in a Teflon vessel with 8 mL of 65% HNO_3_ and 2 mL of 30% H_2_O_2_. Then, 0.5 g sediment samples were added to the vessels with 2.5 mL of 65% HNO_3_ and 7.5 mL of HCI [13]. The samples in the vessels were then digested using an optimized microwave method. Then, they were cooled at room temperature. The residues were then dissolved and diluted to 15 mL. The chemicals used for the sample dissolution were of analytical grade. Ultra pure water was used throughout the study [14].

Metal contents of samples (Cr, Ni, Pb, Cd, Mn, Fe, and Zn) were determined by an Inductively Coupled Plasma Optical Emission Spectrometer (ICP-OES, PerkinElmer DV 2000, Waltham, MA, USA). In the ICP-OES analysis, the following wavelength lines were used: Cr 205.6 nm, Ni 231.6 nm, Pb 220.4 nm, Cd 226.5 nm, Mn 293.3 nm, Fe 238.2 nm, and Zn 213.9 nm.

## 4. Results and Discussion

### 4.1. Fish Samples

The minimum, the maximum, and the average ^210^Po concentrations for the mullet are 1.49 ± 0.34 Bq·kg^−1^ (February), 9.42 ± 0.92 Bq·kg^−1^ (October), and 5.00 ± 0.65 Bq·kg^−1^, respectively. The minimum, the maximum, and the average ^210^Pb concentrations for mullet are ND (non-detectable), 1.16 ± 0.31 Bq·kg^−1^ (March), and 0.99 ± 0.30 Bq·kg^−1^, respectively. 

For sea bass, the minimum, the maximum, and the average ^210^Po concentrations are 3.58 ± 0.46 Bq·kg^−1^ (December), 4.71 ± 0.58 Bq·kg^−1^ (March), and 4.06 ± 0.64 Bq·kg^−1^, respectively. For sea bass the minimum, the maximum, and the average ^210^Pb concentrations are ND, 1.47 ± 0.23 Bq·kg^−1^ (February), and 1.12 ± 0.44 Bq·kg^−1^, respectively. Strok and Smodis [15] measured the ^210^Po and ^210^Pb activities as 0.37 ± 0.07 Bq·kg^−1^ and 2.85 ± 0.48 Bq·kg^−1^, respectively. The researchers stated that ^210^Pb activity concentrations for marine species are usually lower than ^210^Po activity concentrations. However, they found an exception to this for sea bass. They concluded that it is due to the terrestrial feeding habit in mariculture. In Portugal, Malta et al. [16] studied the radioactivity levels for sea bass. They found the mean ^210^Po activity concentrations as 13 ± 7, 3.8 ± 3.1, 0.53 ± 0.52 Bq·kg^−1^ in liver, gonads, and muscle of mullets, respectively. They determined the mean ^210^Pb activities in liver, gonads, and muscle of mullets as 1.39 ± 0.87, 0.32 ± 0.34, and 0.10 ± 0.09 Bq·kg^−1^.

For eel, the minimum, the maximum, and the average ^210^Po concentrations are 4.05 ± 0.84 Bq·kg^−1^ (May), 12.56 ± 1.62 Bq·kg^−1^ (March), and 6.51 ± 0.91, respectively. For eel, the minimum, the maximum, and the average ^210^Pb concentrations are ND, 2.66 ± 0.34 Bq·kg^−1^ (February), and 1.58 ± 0.27, respectively. Al-Masri et al. [17] found the ^210^Po and ^210^Pb concentrations for eel in Syria as 1.03 ± 0.003 Bq·kg^−1^ and 0.005 ± 0.001 Bq·kg^−1^, respectively. 

Cr, Ni, Pb, Cd, Mn, Fe, and Zn levels in the fish samples measured and are given in Table 1 with data from around the world. According to Turkish Food Regulations, the maximum Cd, Pb, and Zn concentrations should not exceed 0.05, 0.3, and 50 mg·kg^−1^, respectively. The observed values in this study do not exceed the given limits. The high concentrations can be linked to the industrial facilities on the Büyük Menderes River, which is the largest water source for Lake Bafa.

The dose values are presented in Table 2. The calculated annual effective ingestion dose for humans due to ^210^Po from the consumption of the lake fishes ranges from 0.011 to 1.169 µSv·y^−1^. It is clear that the highest dose value (1.169 µSv·y^−1^) was observed of mullet. According to the UNSCEAR (United Nations Scientific Committee on the Effects of Atomic Radiation) [18] report, the effective dose equivalent of ^210^Po in areas of normal background radiation is approximately (130 µSv·y^−1^). This dose value is higher than the doses that were found in this study. 

### 4.2. Surface Sediment

The points where the surface sediment samples collected are shown in Figure 2. The average ^210^Po concentrations for surface sediment samples in summer, autumn, winter, and spring are 113 ± 9 Bq·kg^−1^, 128 ± 9 Bq·kg^−1^, 135 ± 9 Bq·kg^−1^, and 134 ± 9 Bq·kg^−1^, respectively. 

The average ^210^Pb concentrations for surface sediment samples in summer, autumn, winter, and spring are 56 ± 6 Bq·kg^−1^, 58 ± 7 Bq·kg^−1^, 111 ± 13 Bq·kg^−1^, and 46 ± 5 Bq·kg^−1^, respectively.

**Figure 3:**
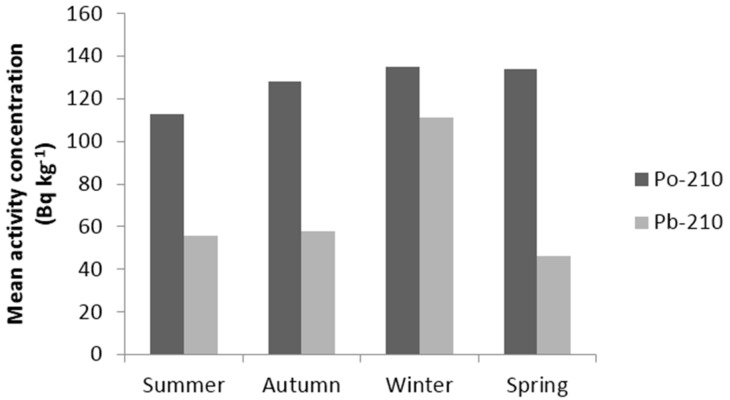
The seasonally mean value of ^210^Po and ^210^Pb in surface sediment.

The average ^210^Po and ^210^Pb values are given in Figure 3 seasonally.

There are significant seasonal differences in ^210^Pb concentrations. The highest ^210^Pb concentrations were observed in the winter. The sampling station is under the influence of Mediterranean climate with cool wet winters and warm to hot, dry summers. Most of the precipitation falls during the winter months. It is believed that the elevated concentrations of ^210^Pb in sediments in winter could be linked to high atmospheric deposition of ^210^Pb during this season [25]. 

The ^210^Po:^210^Pb activity ratios in surface sediments samples varies between 0.85 and 4.48 as shown in Table 3, with a mean value of 2.14. There are significant differences in ^210^Po and ^210^Pb concentrations with respect to different seasons. These differences could be due to the local pollution sources as well as variations in the riverine and rainfall regimes. 

### 4.3. Core Sample

The minimum, the maximum, and the average ^210^Po concentrations in core samples were found to be 32 ± 1 Bq·kg^−1^ (at 33 cm), 362 ± 20 Bq·kg^−1^ (at the surface), 116 ± 5 and Bq·kg^−1^, respectively. The minimum, the maximum, and the average ^210^Pb concentrations in core samples were found to be 14 ± 2 Bq·kg^−1^ (at 34.2 cm), 201 ± 18 Bq·kg^−1^ (at 1.2 cm), and 71 ± 8 and Bq·kg^−1^, respectively.

**Figure 4:**
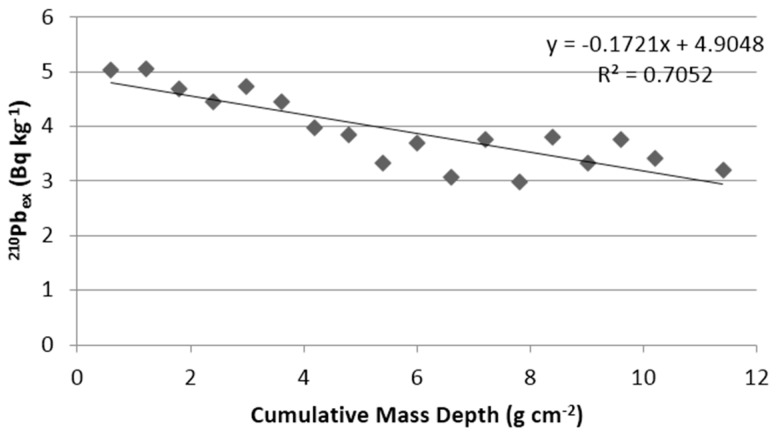
^210^Pb_ex_ activity concentrations on a logarithmic scale (ln) versus cumulative mass depth.

Figure 4 shows ^210^Pb_ex_ activity concentrations on a logarithmic scale versus cumulative mass depth. Activity concentration of excess ^210^Pb varies exponentially along the sediment profile. The respective maximum concentration (159 ± 18 Bq·kg^−1^) was measured in the 1.2 cm sediment layer. The equilibrium point is at 12.6 cm. The ^210^Pb concentration in the core sample decreases with depth, so the ^210^Pb flux of the sediment is constant. The age of the sediment layers was calculated with the CRS model. The results are given in Table 4. The deepest sediment layers, at depths between 31.8 and 33 cm, had been deposited around the beginning of the 1900s.

In the study, the sediment mass accumulation rate was found to be 3.27 g·m^−2^·day^−1^ (0.119 g·cm^−2^·y^−1^). The sedimentation mass accumulation rate in Lake Bafa is relatively lower than Lake Hachirogata and Lake Erie. Jin et al. [26] found the sediment mass accumulation rate in Lake Hachirogata 0.43 g·cm^−2^·y^−1^ and 0.45 g·cm^−2^·y^−1^ in two core samples, and Klump et al. [27] determined the accumulation rate in an average of 0.23 g·cm^−2^·y^−1^ for Lake Erie.

The heavy metal concentrations in vertical profile of samples from the core are given in Table 5. For all the metals investigated, there is a peak value at 16 cm and below this point the concentrations are decreasing with depth. Except Fe and Zn, the highest and lowest concentrations are at 16 cm and 4.5 cm, respectively. This coincides with the beginning of industrialization in the region. The measured concentration of Fe and Zn are in the range 3.11%–4.13%, 68–90 ppm, respectively. The Zn concentrations in the core sample are lower than the ERL (effects range low) and ERM (effects range median) limits [28] (Table 6). High Fe and Zn concentrations were observed at 3 and 6 cm. The measured concentration of Cd and Pb are in the range 0.09–0.20 ppm, 8.3–19.1 ppm, respectively. Cd and Pb concentrations show a similar pattern. The concentrations of both metals are below the ERL limits. The measured concentration of Cr and Ni are in the range of 214.4–288.3 ppm, 297.2–457.9 ppm, respectively. While the Cr concentrations are between the ERL and ERM limits, Ni concentrations are higher than the ERM limit.

## 5. Conclusions

In this study, the distribution of natural radionuclides and certain heavy metals were determined in Lake Bafa via sediment and biota samples. Surface sediment samples were taken seasonally. The highest ^210^Pb concentrations were observed in winter because of the Mediterranean climate and high rainfall regime. Most of the precipitation falls during the winter months. This indicates that the main source of ^210^Pb is from atmospheric deposition through precipitation. In addition, the sediment mass accumulation rate was found to be 3.27 g·m^−2^·day^−1^ (0.119 g·cm^−2^·y^−1^) from the core sample. The lake is the delta progradation of the Büyük Menderes River, so it could be considered a sediment trap area. The heavy metal concentrations in the vertical profile of samples from the core were also observed. The measured concentration of Zn, Pb, Cd, and Cr are between the ERL and ERM limits, while Ni concentrations were higher than the ERM limit. Although nickel does not accumulate in fish, plants, or animals, it does accumulate in soils and sediments and may ultimately have an adverse effect on water quality [29].

The ^210^Po and ^210^Pb concentrations were obtained in the range of other studies around the world. The differences in the radionuclide concentration can be explained by the feeding habitat. Mullet is a pelagic species. They feed on epiphytic algae, detritus, and small benthic or planktonic organisms. Sea basses feed on small fish, polychaetes, cephalopods, and crustaceans. Eels are predators. In winter and spring, the maximum ^210^Po concentrations were observed in eel fish samples. In addition, the highest dose value (1.169 µSv·y^−1^) was observed for sea bass and the annual effective ingestion dose for humans since ^210^Po from the consumption of the lake fishes were calculated between 0.011 and 1.169 µSv·y^−1^. ^210^Po concentrations were highest in sea bass due to feeding habits. Aarkrog et al. [30] calculated that the individual doses ranged from 5.1 to 9.1 µSv·y^−1^. Pollard et al. [31] and Nielsen et al. [32] estimated doses of 19 µSv·y^−1^ and 700 µSv·y^−1^, respectively. Jia et al. [33] calculated exposure values between 50 and 200 µSv·y^−1^. The maximum observed Cd, Pb, and Zn concentrations do not exceed 0.05, 0.3, and 50 mg·kg^−1^, respectively, according to Turkish Food Regulations. Although contaminants measured in fish and sediments in this study do not pose a significant risk to human health, it is important to monitor the lakes from an ecological point of view.

## Figures and Tables

**Table 1:** Heavy metal concentrations (mg·kg^−1^) in fish samples in the literature.

Species	Area	Cr	Ni	Pb	Cd	Mn	Fe	Zn	Reference
Seabass (*D. labrax*)	Aegean Sea 1		ND	ND	ND		5.49	6.38	Kalantzi et al. [19]
	Aegean Sea 2		0.19	ND	ND		ND	5.39	Kalantzi et al. [19]
	Portugal		0.0007	0.000038	0.0062			11.24	Cid et al. [20]
	Spain			0.004	0.002				Olmedo et al. [21]
	**Lake Bafa**	**0.42**	**0.72**	**0.12**	**0.01**	**1.28**	**8.71**	**8.53**	**This study**
Mullet (*L. ramada*)	Antalya	ND	ND			0.28	7.13	12.28	Uysal et al. [22]
	River Guadalguivir		0.07–0.26	0.03–0.06	0–0.003	0–0.97	5.6–15.1	4.4–21.5	Blasco et al. [23]
	**Lake Bafa**	**0.21**	**0.78**	**0.14**	**0.03**	**1.0**	**8.26**	**13.6**	**This study**
Eel (*A. anguilla*)	Portugal (Sao Jacinto)		0.200–0.394	0.0541–0.0783	0.0109–0.0411			19.7–23.1	Cid et al. [20]
	Portugal (Torreira)		0.157–0.187	0.0440–0.0564	0.0109–0.0423			14.0–21.7	Cid et al. [20]
	Portugal (Barra)		0.387	0.0526	0.0089			20.8	Cid et al. [20]
	Portugal (Gafanha)		0.200	0.0744	0.0258			20.7	Cid et al. [20]
	Spain (Bacuta)	0.209	0.016	0.09		14.1	4.93	11.4	Usero et al. [24]
	Spain (Liebre)	0.143	0.015	0.05		13.1	4.11	11.0	Usero et al. [24]
	Spain (San Carlos)	0.364	0.015	0.05		6.8	5.89	10.1	Usero et al. [24]
	Spain (San Juan)	0.368	0.020	0.03		4.71	5.19	13.0	Usero et al. [24]
	**Lake Bafa**	**0.84**	**1.43**	**0.17**	**0.04**	**2.61**	**11.36**	**29.9**	**This study**

**Table 2:** Annual dose due to consumption of fish species contaminated by ^210^Po.

Fish Species	Dose (µSv·y^−1^)
Seabass (*D. labrax*)	0.208
Mullet (*L. ramada*)	1.169
Eel (*A. anguilla*)	0.011

**Table 3:** The ^210^Po:^210^Pb ratios in surface sediments from all sampling points.

Sample	^210^Po:^210^Pb			
	Spring	Summer	Autumn	Winter
1	3.38	2.36	1.40	1.07
2	2.71	2.34	2.05	1.01
3	2.42	2.21	1.69	1.16
4	2.16	1.82	2.61	1.15
5	3.25	2.94	2.54	1.17
6	3.36	2.72	2.05	0.85
7	4.48	1.65	2.02	1.20
8	4.37	1.60	1.62	1.15
9	1.93	2.00	2.62	1.24
10	2.25	1.65	2.93	1.63
11	1.69	1.74	1.98	1.38
12	4.28	1.49	2.62	1.55
13	2.28	1.59	3.66	1.21
14	3.04	2.02	3.10	1.33
15	3.58	1.78	1.80	1.38

**Table 4:** Vertical distribution of ^210^Pb in dated layers.

Depth (cm)	Cumulative Depth (g·cm^−2^)	^210^Pb_ex_ (Bq·kg^−1^)	Year
0.6	0.45	150.66	2011
1.2	0.83	158.91	2008
1.8	1.21	107.75	2006
2.4	1.61	84.74	2005
3.0	2.03	114.96	2002
3.6	2.45	85.90	2000
4.2	2.88	54.10	1998
4.8	3.30	46.80	1997
5.4	3.72	27.50	1995
6.0	4.14	40.77	1994
6.6	4.50	21.30	1992
7.2	4.90	43.20	1990
7.8	5.31	19.75	1989
8.4	5.69	44.80	1987
9.0	6.06	27.77	1985
9.6	6.43	42.26	1983
10.2	6.81	29.81	1980
11.4	7.54	24.48	1978
12.6	8.33	0.00	1976
13.8	9.09	0.00	1974
15.0	9.77	18.61	1972
16.2	10.47	47.66	1970
17.4	11.16	0.26	1966
18.6	11.83	0.00	1964
19.8	12.48	11.96	1962
21.0	13.12	0.00	1959
22.2	13.78	0.00	1957
23.4	14.46	0.00	1954
24.6	15.14	4.75	1951
25.8	15.84	8.11	1947
27	16.51	11.73	1943
28.2	17.21	0.00	1937
29.4	17.86	6.73	1935
30.6	18.55	2.78	1928
31.8	19.27	13.46	1919
33	19.94	0.00	1905

**Table 5:** The heavy metal concentrations in vertical profile of core samples.

Depth (cm)	Heavy Metal Concentrations (ppm)						
	Fe	Zn	Mn	Pb	Cd	Cr	Ni
1.50	36,266.95	78.84	703.08	17.28	0.18	259.20	307.80
3.00	41,345.43	89.88	759.70	12.84	0.13	267.50	334.91
4.50	35,972.55	78.20	644.00	8.28	0.09	214.36	297.16
6.00	38,944.19	84.66	686.46	14.28	0.15	239.70	389.64
7.50	36,515.36	79.38	685.02	13.72	0.14	222.46	393.96
9.00	32,264.89	70.14	713.42	16.03	0.17	235.47	393.79
13.00	31,050.47	67.50	676.80	13.50	0.14	250.20	420.30
16.00	35,107.73	76.32	779.10	19.08	0.20	288.32	457.92
19.00	33,465.51	72.75	744.96	12.61	0.13	249.29	459.78
26.00	31,423.08	68.31	721.71	8.91	0.09	244.53	346.50

**Table 6:** ERL (effects range low) and ERM (effects range median) limits for metals.

Metal	ERL ^a,b^	ERM ^a,b^
Cadmium (Cd)	1.2	9.6
Chromium (Cr)	81	370
Lead (Pb)	47	220
Nickel (Ni)	21	52
Zinc (Zn)	150	410

**^a^** Units are µg/g dry sediment, equivalent to ppm. **^b^** ERL values as the lowest concentration of a metal that produced adverse effects in 10% of the data reviewed. Similarly, the ERM designates the level at which half of the studies reported harmful effects [28].
